# Effects of Puberty on Blood Pressure Trajectories — Underlying Processes

**DOI:** 10.1007/s11906-023-01241-9

**Published:** 2023-04-18

**Authors:** Małgorzata Wójcik, Jerzy B. Starzyk, Monika Drożdż, Dorota Drożdż

**Affiliations:** 1 Department of Pediatric and Adolescent Endocrinology, Chair of Pediatrics, Pediatric Institute, Jagiellonian University Medical College, Kraków, Poland; 2 Department of Pediatric Nephrology and Hypertension, Chair of Pediatrics, Pediatric Institute, Jagiellonian University Medical College, Kraków, Poland

**Keywords:** Arterial hypertension, Blood pressure, Puberty, Adolescents

## Abstract

Puberty is a complex process leading to physical, sexual, and psychosocial maturation. The changes in morphology and organ function during puberty also affect blood pressure (BP) regulation, and as a consequence (BP) values change noticeably, reaching values often higher than after reaching full maturity. In children entering puberty, BP, especially systolic, increases and then reaches adult values by the end of puberty. The mechanisms responsible for this process are complex and not fully understood. Sex hormones, growth hormone, insulin-like growth factor-1, and insulin, whose production increases during puberty, significantly regulate BP through complex and overlapping mechanisms. During puberty, the incidence of arterial hypertension also increases, especially in children with excess body weight. The present paper presents the current state of knowledge regarding the influence of processes occurring during puberty on blood pressure.

## Introduction

Puberty is a complex process leading to physical, sexual, and psychosocial maturation [1]. It is a unique period in the life of every human being, in which the maturation processes of all organs and systems are being finalized. Transformation processes are associated with physiological changes that are reflected in the results of anthropometric, biochemical, and imaging tests. Since puberty is a relatively long-term process and very individual, it is often difficult to interpret the results of these tests, as the obtained values while correct for this period may be very similar or identical to the values considered abnormal in other periods of life. During puberty, differences in the structure and function of organs and systems between the sexes become clear and visible. For these reasons, for many parameters it has not been possible to determine reference values at all, and for others the interpretation requires taking into account many variables, such as body height or advancement of sexual maturity. Observational studies indicate that during puberty, blood pressure values change noticeably, reaching values often higher than after reaching full maturity [2, 3•, 4•]. In a systematic review and meta-analysis of 47 articles published between 2000 and 2015, Song et al. found a pattern of hypertension prevalence based on pubertal stage and age [5]. The prevalence of childhood hypertension started to increase rapidly from the onset of puberty and reached the peak level at the end of puberty. At the age of 14 years, it was 7.89%, compared to 4.32% and 3.28% at the age of 6 and 19 years respectively [5].

### Physiology of the Puberty

The basis of changes occurring during puberty is the process of gonadal maturation (gonadarche), involving the ovary or testes. In parallel, independently, the process of adrenal maturation (adrenarche) takes place. Finally, both phenomena cause an increase in the production of sex hormones leading to biological changes in the body. Gonadarche is initiated by cells of the hypothalamus that secrete gonadotropin-releasing hormone (GnRH), and that subsequently increases the pulsative release of luteinizing hormone (LH) and follicle-stimulating hormone (FSH). In boys, LH stimulates testosterone production and FSH supports sperm maturation. In girls, both FSH and LH stimulate ovary production of estrogen, progesterone, and testosterone. FSH initiates follicular growth, specifically affecting granulosa cells. Adrenarche can occur separately from the onset of pituitary secretion of LH and FSH. It results from changes in the secretory response to adrenocorticotropin (ACTH) leading to the dehydroepiandrosterone sulfate (DHEAS) rise. Adrenal androgens normally contribute to the onset of sexual pubic hair (pubarche) and sebaceous and apocrine gland development [6•]. Control of the timing and onset of puberty remains an enduring biological mystery. Both genetic and environmental factors play substantial roles in pubertal onset. Research published in the last two decades allowed us to determine the contribution of several neuropeptides in the initiation of the pulsatile secretion of GnRH. Kisspeptin is the main factor stimulating GnRH secretion. Its action is modified by opposing or permissive signaling of two other peptides: neurokinin B and dynorphin [7]. The entirety of this puberty-initiating system is described by the acronym KNDy (Kisspeptin-Neurokinin B- Dynorphin) [8]. This pulsatile production of GnRH is followed by a rise in LH and, consequently, in gonadal steroids. Much evidence indicates that the initiation of KNDy action is influenced by genetic and epigenetic factors, including, for example, nutritional status, leptin concentration, stress exposure, and others [7]. Less is known about the factors leading to the onset of adrenarche that is characterized by a change in the pattern of the adrenocortical steroid response to ACTH due to apparent increases in 17,20-lyase and sulfotransferase activity and a decrease in 3ß-hydroxysteroid dehydrogenase (3ßHSD) activity [6•]. These changes in enzyme activities result in increased synthesis of adrenal androgens: Dehydroepiandrosterone (DHEA), and subsequently its metabolite DHEAS.

### The Specificity of Interpreting BP Values During Puberty

There is no doubt that normal blood pressure (BP) values change with the age of the child and the child’s body size increase. In addition, during puberty, the value of BP is influenced by the activity of hormones whose secretion increases at this time. Therefore, in the interpretation of the results of BP measurements, special standards that adjust for the changes in normal BP values over childhood should be followed for both office and 24-h ambulatory BP [2]. This approach was proposed and accepted for the first time in the 1970s and, despite various modifications and confusion is still valid today [9–12]. Since sexual development is a very individual process, children at the same chronological age may be at different stages of puberty. However, the synchronization of aging, accelerated growth, an increase of body weight, and pubertal development has led to inconsistent results on which of these factors is the most important [13, 14]. The few studies conducted so far in this field have proven that the advancement of puberty is associated with changes in BP values [15, 16]. In a population of 7–12-year-old children, Li et al. showed that puberty advancement is significantly more associated with blood pressure value than chronological age [4•]. In children who presented with puberty onset, the systolic and diastolic BP values were significantly higher than levels in those who were prepubertal and in the same age group. Mean values of BP in the age groups from 7 to 12, depending on whether the children were prepubertal or pubertal, differed by 2.5 to 7 mmHg for SBP and 2 to 5 mmHg for DBP. These differences in BP between pubertal and prepubertal children were statistically significant even after adjusting for children’s age, sex, body mass index, taste preference, and family history of hypertension (coefficient for SBP 3.84 [95%CI 2.92,4.75], for DBP 2.24 [95%CI 1.39,3.09] [4•]. Moreover, a few studies showed that children with higher BP present with more advanced bone maturation during puberty [17–19]. This observation is important as bone age is the parameter of auxological assessment that best reflects the advancement of biological development.

It has not been determined how to interpret blood pressure values depending on the stage of puberty so far. Considering the remarks above, when interpreting BP values, one should not refer only to the child’s chronological age. Incorporation of height into the generation of BP reference values may adjust somewhat for pubertal status as growth velocity and height percentile position correlate with puberty more than chronological age [9]. For the same reason, use of thresholds for both sexes beginning at the age of 13 years as recently recommended in the American Academy of Pediatrics clinical practice guidelines is problematic. Please note that this group may include both fully sexually mature (stage V) and prepubertal children, as it is perfectly normal for puberty to begin at any point between the ages of 8 and 13 in girls and 9 and 14 in boys.

### Hormonal Changes During Puberty and Their Impact on Blood Pressure

#### Androgens

The effect of androgens on BP in adolescence is not clear. Research in this area is particularly difficult because androgens of adrenal and ovarian origin are aromatized to estrogens, including in peripheral tissues. For this reason, even in experimental studies, it is extremely difficult to separate the effects of the androgens themselves. The increase of the production of androgens DHEA, and DHEAS by the adrenal glands at about 6–7 years of age is one of the first essential elements of sexual maturation (adrenarche) [6•]. Subsequently, the gonadal axis is activated, increasing the synthesis of androgens in the gonads in both sexes, but to a greater extent of course in boys. In conjunction with that increase, there is an increase in BP values [20]. Various epidemiological, clinical and experimental studies have indicated that androgens may be important determinants of sex-specific differences in arterial blood pressure. Sexual dimorphism in blood pressure develops in puberty and persists during adulthood [21–23]. However, it is unclear whether DHEA and DHEAS are associated with BP regulation independently or through conversion to other androgens or even to estrogens [24, 25]. What more, although epidemiological and experimental studies have shown that androgens are involved in the regulation of blood pressure, the pathophysiological mechanism is not well understood. An additional difficulty is the fact that the effect of androgens on blood pressure regulation is probably species-specific, therefore not all results obtained in animal studies can be applied to humans. The effect of androgens can also be variable, on the one hand, they can increase and on the other hand, they can lower blood pressure in various mechanisms [21, 26]. While acute administration of DHEA or testosterone seems to decrease vascular tone, the long-term net effect of androgens appears to be vasoconstriction, via both direct and indirect pathways. It has been shown that in an animal model, testosterone promotes epinephrine and norepinephrine synthesis in the adrenal medulla, and therefore has an indirect vasoconstrictive effect [26]. It is postulated that testosterone might be a trigger for an increase in secretion of endothelin-1 by endothelial cells, and an increase of thromboxane A2 receptor expression, both known to have strong vasoconstrictive effects [27–29]. It has been shown that testosterone increases plasma renin activity, and enhances synthesis of angiotensinogen and angiotensin II [30, 31]. The degree to which aldosterone release is influenced by androgens has not been fully clarified yet [32]. But, interestingly, the epithelial sodium channel (ENaC), the target for aldosterone action, seems to be regulated by androgens as well, because testosterone may stimulate the expression of the ENaC-subunit [33]. So there is evidence that androgens stimulate processes leading to the increase of BP in many different ways. Studies conducted in children during puberty revealed a positive association between circulating testosterone and SBP in boys and girls [34]. It was found, not surprisingly, that circulating testosterone levels in boys were higher than values in girls, which may explain differences in BP values between the sexes that are marked during puberty [34]. Convincing evidence of the effect of adrenal androgens on blood pressure also comes from studies of patients with inborn hyperandrogenism such as congenital adrenal hyperplasia. Their blood pressure values are higher than those of healthy peers, and correlate with the degree of hyperandrogenism and even with the genotype [35–37].

#### Estrogens

Estrogens are the main hormones responsible for the effects of puberty in girls. In the regulation of BP in adolescent girls and women, the balance between estrogens and androgens seems to be of particular importance. Estradiol, the main hormonal product of the ovary, has a pressure-lowering effect and is known to have a protective role regarding the cardiovascular and renal system. The increase in estrogen concentration in girls during puberty may therefore be responsible for lower blood pressure than in boys during this period. Indirect evidence comes from observations of patients with polycystic ovary syndrome in whom estrogen deficiency is relative and androgen overproduction predominates, and patients with Turner’s syndrome who have absolute estrogen deficiency. Adolescents with polycystic ovary syndrome have been shown to have significantly higher blood pressure on 24-h ambulatory blood pressure monitoring than their peers with similar anthropological parameters but without hyperandrogenism [38]. Patients with Turner syndrome have higher blood pressure measurements compared to published population standards, as evidenced by the shift to the right of both the systolic and diastolic BP. But interestingly, patients on estrogen therapy had no significant difference in mean manual and 24 h BP recordings compared to the group without estrogen replacement [39]. Very little is currently known about the exact mechanisms of action of estrogens on BP. It seems, however, that the blood pressure lowering effect is a kind of ‘net’ effect resulting from their participation in various vasorelaxation and vasoconstriction processes [40]. Estradiol stimulates synthesis of the angiotensinogen in the liver. That subsequently may increase RAAs activity, and sodium and fluid retention. But it is also known that by affecting the endothelium in blood vessels, estradiol can have a vasorelaxing effect [41]. Estrogen receptors have been identified in the endothelium, intima, and adventitia, as well as in vascular smooth muscle of several species, including humans [42, 43]. Estrogens have been demonstrated to enhance endothelial-dependent relaxation by increasing nitric oxide production and release [44]. Furthermore, estrogens have antioxidant properties and prevent nitric oxide degradation, increasing, consequently, nitric oxide availability [40]. Estrogens also inhibit vascular smooth muscle cell proliferation and neointima formation in response to arterial injury in animal models [45]. On the other side, there is evidence from animal studies that chronic exposure to low levels of estradiol may increase mean arterial pressure, and this is associated with increased endothelin 1 activity [46]. Moreover, at physiological concentrations, estrogen increases proinflammatory cytokines like interleukin 1, interleukin 6 and TNFα [47]. However, due to the protective effect on the endothelium, estrogens at physiological concentrations have a beneficial effect on BP regulation.

#### Progesterone

As puberty progresses in girls, the production and biological role of progesterone increases. Progesterone has a natriuretic and blood pressure lowering effect, mainly due to its competition with mineralocorticoids for the type I corticosteroid receptor [48]. Progesterone leads to sodium loss and a compensatory increase in renin secretion and plasma renin activity. Therefore, the rise in plasma renin activity, angiotensin II and aldosterone in the second half of the menstrual cycle is caused by progesterone [49]. The latter phenomenon, however, is a compensatory effect and has no significant effect on BP.

### Growth Hormone and Insulin-Like Growth Factor-1

Growth hormone (GH) and its leading effector, insulin-like growth factor 1 (IGF-1) are the main modulators of body longitudinal growth [50•]. Their secretion and activity significantly increase during puberty, leading to an increase in growth rate (pubertal growth spurt), and enabling the achievement of final height. Growth hormone and IGF-1, not only regulate somatic growth, but also help control a number of physiological processes. It also has been reported that GH and IGF-1 directly or indirectly regulate BP [51, 52]. Evidence indicates that GH plays a significant role in angiogenesis, contributing to regulation of vascular growth and function [53]. There is clear evidence that receptors for GH and IGF-1 are expressed in the vascular endothelium and myocardium [54–57]. GH plays a key role for the development of a normal heart during fetal development, and plays a positive role in maintaining the structure and function of the normal adult heart, by stimulating cardiac growth and heart contractility, and also the structure of a normal vascular endothelium [54]. Growth hormone and IGF-1 are the most potent angiogenetic agents. Experimental and clinical studies suggest that GH has an anti-natriuretic effect which appears to be due to direct action in the kidney or indirectly through IGF-1 or insulin [58]. Interestingly, GH is also produced by endothelial cells, and endothelium-derived GH stimulates the proliferation, migration, survival, and capillary formation of endothelial cells in an autocrine manner [59]. The relationship between BP and IGF-1 is dependent on, or related to, IGF-1 concentrations, as an expression of direct or reverse causality. Low IGF-1 bioavailability (associated with aging and vascular deterioration), resistance to IGF-1, and the complex interplay between IGF-1 and other vasoactive hormones could mask the vasoprotective functions of IGF-1 [60]. Therefore, the effect of GH on BP regulation in adolescence is quite complex and is related to the activity of other hormones, including sex hormones. Studies conducted in patients with growth hormone deficiency, during substitution treatment and in the case of its endogenous overproduction indicate a complex mechanism of action, in which it is important whether the concentrations of GH and IGF-1 are normal, too low or too high. Patients with growth hormone deficiency in childhood present with lower BP, while due to premature aging of the blood vessels, and the increase of vascular stiffness in adulthood, they often develop hypertension. Giving patients with GH deficiency adequate substitution treatment significantly lowers blood pressure and reduces cardiovascular risk [61–63]. It is suspected that some vasoactive effects of GH may stem from central effects with a subsequent decrease in sympathetic outflow to vascular smooth muscle [64]. This effect on sympathetic activity in vascular smooth muscle has been confirmed by a study in adults receiving GH replacement therapy [64]. In blood vessels, GH and IGF-1 activate the nitric oxide (NO) system and thereby reduce vascular tone [65, 66]. Another possible mechanism for the GH/IGF-I influence on vascular tone involves the regulation of gene expression of the vascular smooth muscle K_ATP_ channel [67]. At the other end of the pathological spectrum are patients with disorders related to the excess of endogenous GH and IGF-1 — giantism and acromegaly. They may develop hypertension, and the contribution of GH and IGF-1 excess to the genesis of the hypertension is supported by the lowering of blood pressure observed concomitantly with reduction in GH levels after successful therapy [58]. The exact mechanisms underlying these effects are not understood. While a direct effect of excess GH and IGF1 through the mechanisms described above is suggested, many experts believe that an indirect effect through the development of insulin resistance and hyperinsulinism is crucial [58, 68].

### Insulin and Insulin Resistance

**Fig. 1:**
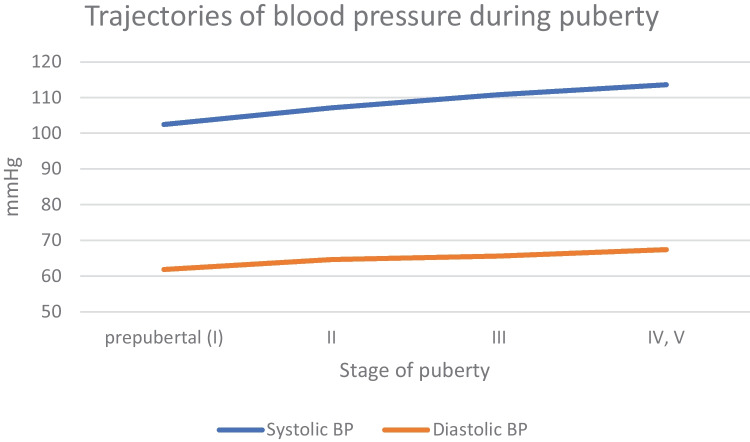
Trajectories of blood pressure during puberty (based on data by Li et al.; Li Y, Dong Y, Zou Z, Gao D, Wang X, Yang Z, et al. Association between pubertal development and elevated blood pressure in children. The Journal of Clinical Hypertension. 2021 Aug 3;23(8):1498–505.)

The relationship between insulin, insulin resistance and blood pressure is quite well known in patients with obesity [69, 70•]. Puberty is associated with a marked decrease in insulin sensitivity. In healthy adolescents, there is a nadir in insulin sensitivity in mid-puberty, and then it recovers at puberty completion [71]. Studies on the natural trajectory of insulin sensitivity in puberty suggest that insulin sensitivity decreases immediately at pubertal onset, nadirs around Tanner stage 3 and then recovers to prepubertal levels in Tanner stage 5 [72] (Fig. 1). However, there is evidence that insulin resistance does not resolve in youth who are obese going into puberty and may result in increased cardiometabolic risk [71]. Data on differences in insulin sensitivity between girls and boys are contradictory and inconclusive [71]. Insulin resistance resulting from reduction in phosphoinositide 3-kinase/protein kinase B signaling, causes a reduction in nitric oxide synthesis that leads to the impairment of tubule-glomerular feedback, subsequent hyperfiltration, and sodium retention [74]. The increase in plasma sodium also stimulates the thirst center, leading to water intake and secretion of arginine vasopressin, resulting in water retention [73]. Although these mechanisms restore plasma sodium to its previous levels, the resulting increase in extracellular volume stimulates other compensatory mechanisms involved in the autoregulation of vascular resistance [72, 74]. Additionally, hyperinsulinemia activates the sympathetic nervous system. This effect appears to be mediated by stimulation of proopiomelanocortin neurons in the arcuate nucleus of the hypothalamus and activation of glutamatergic neurons and melanocortin 4 receptor [75]. That effect on target relevant tissues, including the kidneys and blood vessels raise BP value [69, 74, 76]. Despite the above-mentioned evidence, it is difficult to clearly separate the role of insulin from other effects that increase blood pressure in obese patients. Doubts arise, especially when we analyze the results of studies of patients with insulinoma who do not have hypertension despite severe hyperinsulinemia [77]. Recent studies show that hyperinsulinemia may not initiate obesity hypertension itself, but rather decreased insulin sensitivity which is a trigger for hyperglycemia and dyslipidemia. In that way, it may contribute to progressive vascular and kidney injury which, over the long term, can exacerbate hypertension and lead to further target organ injury and development or progression of hypertension [74].

### The Effect of Weight Gain, Overweight and Obesity During Puberty on Blood Pressure

**Fig. 2:**
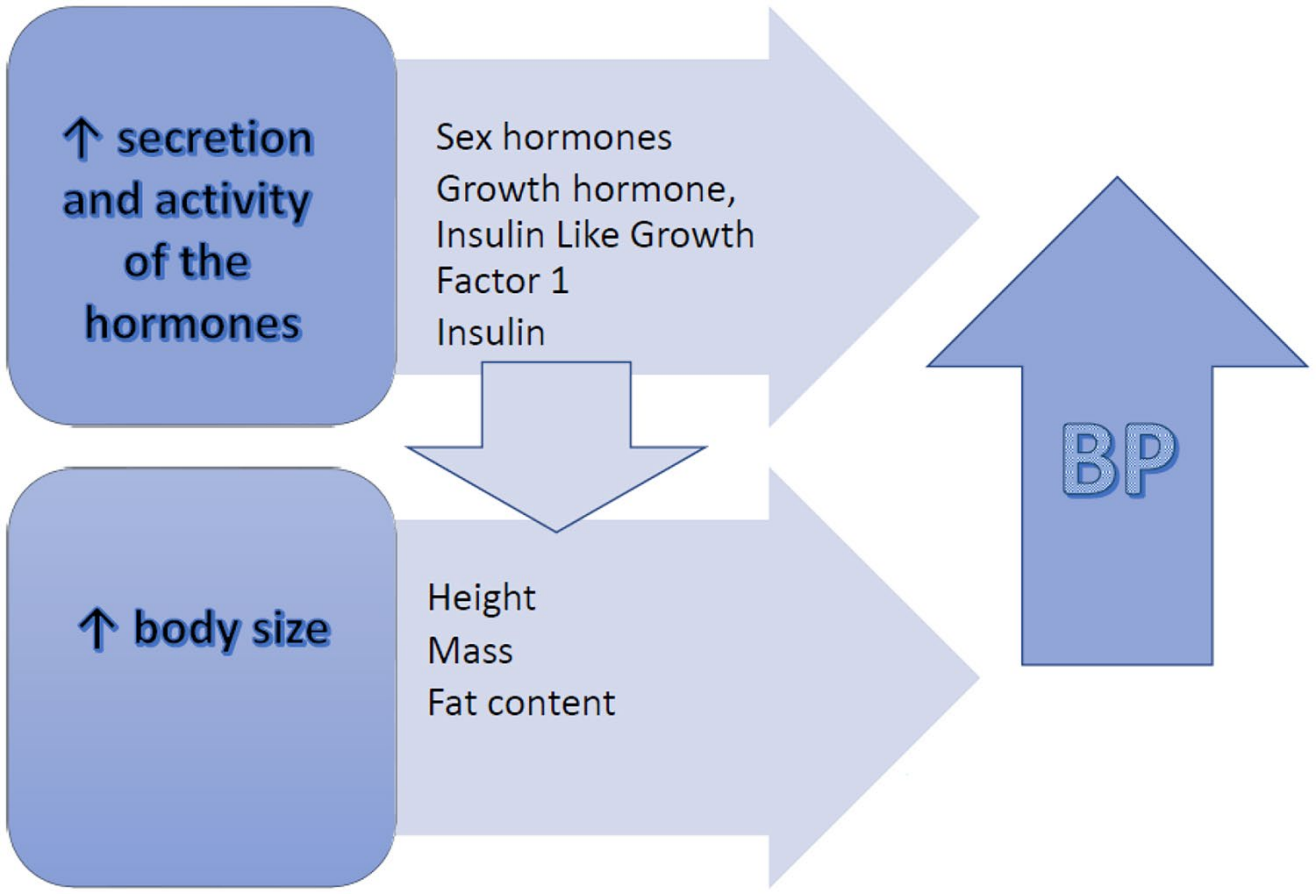
Impact of pubertal changes on blood pressure (BP)

In recent decades, there has been significant weight gain in children and adolescents in all parts of the world. The prevalence of obesity and overweight in subsequent generations has increased significantly, reaching epidemic proportions [78]. The excess weight gain related to overnutrition can affect growth patterns, bone maturation and pubertal development (Fig. 2). Linear growth and bone maturation usually is accelerated in prepubertal children with obesity, however, subsequently there is a reduction of pubertal height gain such that any advantage in linear growth is lost and their final height does not differ from non-obese children [79]. The relationship between obesity and hypertension is well established [80]. Considering the obesity epidemic, there appeared a risk that the normal values and subsequently the thresholds defining hypertension would be higher if new nomograms were created based on studies carried out in more recent years. Before 2017, both US and European guidelines used percentiles derived from the same reference population for the interpretation of BP values in pediatric patients [9, 11, 81]. In 2017, the American Academy of Pediatrics proposed the exclusion of subjects with excess of body weight from the ‘historical’ reference cohort, and created new reference nomograms [9]. These new nomograms redistributed BP values in the pediatric population. Despite the theoretically correct assumption that eliminating children with excessive body weight from the analysis will allow for the development of more reliable data on the distribution of normal blood pressure in children, doubts regarding the necessity for this approach have been raised in several recent studies [82–84]. Resolution of these concerns will require more investigation.

## Conclusions

Physiological changes during puberty affect the regulation of blood pressure. In children entering puberty, BP, especially systolic, increases and then reaches adult values by the end of puberty. The mechanisms responsible for this process are complex and not fully understood. Obesity in adolescence is conducive to the persistence of high values of BP and the development of arterial hypertension.
